# Translational Needs: Informing Tools to Close the Knowledge‐to‐Practice Gap in Suicide Prevention

**DOI:** 10.1002/hpja.70142

**Published:** 2026-01-29

**Authors:** Max Tran, Leicha Stewart, Jose Cuenca, Melinda Benson, Lennart Reifels

**Affiliations:** ^1^ Everymind Newcastle Australia; ^2^ University of Newcastle. Newcastle Australia; ^3^ Centre for Mental Health, Melbourne School of Population and Global Health University of Melbourne Melbourne Australia

**Keywords:** evidence, implementation, knowledge translation, research, suicide prevention

## Abstract

**Objective:**

Suicide is a major global health concern that accounts for approximately 1.4% of all deaths worldwide. Suicide prevention efforts face a number of implementation challenges. Some studies estimate it takes over 17 years for research innovations to be translated into practice. The delayed adoption of research into practice restricts patient benefits and wastes scarce resources. Developing ways to communicate research findings in formats that are accessible and valuable to those working in suicide prevention is crucial. Using a cross‐sectional online survey, this research assessed the translational needs of stakeholders involved in Australian suicide prevention to determine ways to support the meaningful translation and dissemination of research findings. The survey was completed by 93 participants from a range of roles in the mental health and suicide prevention sector.

**Results:**

The study suggests that participants felt confident in their ability to access resources to improve their knowledge and recognised that existing research evidence influenced their work and preferred traditional and professional platforms for knowledge consumption such as conferences or journal articles. The survey found research that is informed and supported by lived experience is highly valued by stakeholders.

**Conclusions:**

Key recommendations for the development of translational tools in the field of suicide prevention include leaning on traditional approaches to knowledge consumption such as educational sessions or webinars, eNewsletters and website content and exploring how lived experience knowledge and expertise can be incorporated into translational tools.

**So What?:**

Disseminating evidence from suicide prevention research in formats preferred by stakeholders, such as plain language summaries, could increase accessibility and reduce the knowledge‐to‐practice gap for successful interventions.

## 1 Introduction

Suicide is a major global health concern that accounts for approximately 1.4% of all deaths worldwide [1]. Despite suicide prevention being named as a national priority in Australia, the interplay of vulnerabilities, risk factors, protective factors and triggers makes effective suicide prevention particularly challenging. In the face of these complexities, good quality evidence about which interventions work and which do not is crucial. There is a growing body of research into effective suicide prevention interventions [2, 3]. However, while many suicide prevention interventions have been shown to be effective, the uptake of effective interventions into practice is slow and some interventions that have been demonstrated to be ineffective are still in common use [4]. Developing accessible ways to communicate research evidence is crucial for using limited resources efficiently and improving outcomes in the sector.

An international commitment to prioritising suicide prevention through research and intervention is important to reduce suicide rates [1, 5]. However, suicide prevention efforts face a number of complex implementation challenges including insufficient collaboration, lack of resources, and limited knowledge of stakeholders about preventive measures [6]. Additionally, there are several gaps in terms of suicide research and evaluation, including a lack of data on priority populations, emergency department presentations, and of routinely collected and timely national data on suicidal thoughts and behaviours [7]. One way to mitigate some of these challenges is to translate existing data, research findings, evidence, and learnings into formats that are accessible and valuable to those working in suicide prevention.

Knowledge translation is the ‘synthesis, exchange, and application of knowledge by relevant stakeholders to accelerate the benefits of global and local innovation in strengthening health systems and improving people's health’ [8]. Translational tools are resources that facilitate moving evidence into practice, assisting with the synthesis, dissemination, exchange, or implementation of knowledge [9]. These tools have often been geared towards patients or clinicians and are commonly discussed in the context of preventative health care and clinical services more broadly. In Australia, there has been a strong shift away from a health‐only approach to preventing the onset of suicidal behaviour by focusing instead on intervening in early distress and connecting people to the support they need [10]. Moving towards this approach requires best practice evidence to be applied in a range of professional roles inside and outside of health. However, little is known about the information and translational needs of the Australian suicide prevention sector.

The issue of translating research findings into practical applications is a well‐established challenge. Some studies estimate it takes over 17 years for research innovations to be translated into practice, and less than 50% will make it into general usage [11, 12]. The delayed or limited adoption of research into practice restricts patient benefits and wastes scarce resources [12]. Therefore, closing this knowledge‐to‐practice gap is a significant issue for suicide prevention. Recent research on the concepts of knowledge translation and the knowledge‐to‐action gap describes the former as a way to reduce the latter. Actionable knowledge is conceptualised as a ‘creative intersection’ or a bridge between important research or evaluation findings that may be inaccessible to suicide prevention stakeholders who are not researchers and action in practice settings [13]. The knowledge‐to‐action gap refers to the gap between emerging evidence‐based research findings and the implementation of these findings to improve suicide prevention outcomes. A number of factors can contribute to the knowledge‐to‐action gap, such as using intervention creators as intervention communicators, introducing interventions before they are ready, and assuming that evidence matters in the decision making of potential adopters [14]. Other challenges particular to the Australian suicide prevention context have been outlined by Kene, Yee and Gimmestad [4] including a lack of training, complexities of decision making, and lacking assessment tools.

The main purpose of providing effective knowledge translation products across the mental health and suicide prevention sector is to equip people who are positioned to implement new knowledge and select evidence‐based interventions with the information they need to make informed decisions. However, not everyone experiencing distress will access services. It is therefore important that communities have access to these resources so that they can benefit from clear, accessible translations of emerging knowledge and evidence in mental health and suicide prevention research.

This research assessed the needs of stakeholders involved in Australian suicide prevention to determine ways to support the meaningful translation and dissemination of research. This study was developed and conducted as part of the LIFEWAYS consortium which has a national mandate to translate suicide prevention research into policy and practice. The study surveyed stakeholders, asking them about the kinds of formats they preferred for receiving up‐to‐date information about novel, evidence‐based suicide prevention interventions and research findings. Translating research knowledge into preferred formats may help to reduce the current timeframe for adoption of interventions which have demonstrated beneficial outcomes, as well as provide valuable learnings to overcome implementation challenges.

## 2 Methods

A primarily quantitative survey was developed as the data collection tool. The survey took approximately five minutes to complete and contained 12 questions, including demographic questions, self‐rated competency questions and questions about preferences for accessing knowledge. It was completed by 93 respondents. The study used a purposive sampling approach, with promotion of the survey taking place across media platforms including LinkedIn, Instagram and Facebook and via email to mailing lists of a number of organisations, state and federal Government departments, PHNs and other suicide prevention organisations. The survey utilised multiple choice questions, checkboxes, Likert scales and additional open text boxes to capture measurable data about information format preferences while offering the flexibility for respondents to expand on their quantitative answers in more detail if they chose to.

Demographic questions were focused on the participants' role and aimed to locate their work within the mental health and suicide prevention sector. The most common roles of participants were providing lived experience, peer support or advocacy (24%), delivering suicide prevention programs or services (20%), funding or commissioning services (19%) and delivering mental health programs or services (14%). Other roles included people working with government policy, conducting research, delivering general health programs and delivering non‐health programs.

Competency questions related to how confident respondents felt to use tools to access new information. The survey used an adapted scale measuring respondents' perceptions of their research and development skills [15]. This was measured using four items from Factor 2 of the Research and Development Culture Index, a non‐standardised instrument for assessing the strength of an organisation's research and development culture. Responses are given on a 5‐point Likert scale with response options strongly disagree (0) to strongly agree (4). Example items include, ‘I understand research terminology’ and ‘I know how practice is influenced by existing research evidence’. Watson et al. [15] reported that Factor 2, personal research and development skills, had a Cronbach alpha of 0.80. In the present study, a Cronbach alpha of 0.84 was observed, indicating good internal consistency.

Further questions explored preferences for content consumption, translational barriers and enablers, and the perceived usefulness of different content and format options. Roses in the Ocean, Australia's national lived experience of suicide organisation, provided an external review of the survey to ensure there was sufficient scope to capture the breadth of possible preferences of those with lived and living experience. The survey was anonymous and administered online using REDCap, a secure interface for data collection. Participation was entirely voluntary, and no questions were compulsory.

Quantitative survey data were analysed by one researcher using Microsoft Excel. Descriptive statistics were calculated for each response and data were presented as frequencies and percentages and means and standard deviations. One missing value in the adapted scale for research and development skills was imputed using the sample mean. There were four qualitative response options that offered participants an opportunity to leave additional feedback that had not been asked about, to note a method or format that hadn't been offered as a response option or to elaborate on barriers or enablers in more detail. Qualitative responses were exported into Microsoft Excel for thematic analysis. A thematic analysis allows a nuanced consideration of related experiences or themes that may not emerge from a word‐based approach such as content analysis. With a sample of over 90 participants, the language and words people use to talk about similar experiences or issues may vary considerably [16].

## 3 Results

The survey was completed by 93 participants from a range of roles in suicide prevention. Respondents worked within Primary Health Networks (25%), non‐clinical services or practices (20%), non‐government organisations (15%), clinical services or practice (13%) and government departments (13%). Within the various settings, 24% of respondents indicated that their role in suicide prevention involved providing lived experience support or advocacy, 20% delivered suicide prevention programs or services, 19% funded or commissioned services and 14% delivered mental health programs or services. Seventy‐four percent of respondents specified that their work supported populations disproportionately affected by suicide.

### 3.1 Research and Development Skills

Participants' self‐reported research and development skills are shown in Table 1.

**TABLE 1:** Participants' self‐reported research and development skills.

	Strongly disagree *n* (%)	Disagree *n* (%)	Neutral *n* (%)	Agree *n* (%)	Strongly agree *n* (%)
I have the skills to use library and learning facilities	1 (1.09)	2 (2.17)	10 (10.87)	50 (54.35)	29 (31.52)
I know how my work is influenced by existing research evidence	1 (1.09)	2 (2.20)	13 (14.29)	52 (57.14)	23 (25.27)
I feel confident about using research in my work	1 (1.09)	3 (3.26)	15 (16.30)	50 (54.35)	23 (25.00)
I understand research terminology	1 (1.09)	4 (4.35)	16 (17.39)	50 (54.35)	21 (22.83)

Each of the four items assessing research and development skills were summed to give a total score for each participant. Participants' total scores ranged between 6 and 16 (M = 12.1; SD = 2.6). Results were calculated across the various primary roles participants indicated they held. Those conducting research (M = 15.2) and delivering general health programs or services (M = 14.0) scored highest, followed by those who fund and commission services (M = 12.3), deliver suicide prevention programs or services (M = 12.0), deliver mental health programs or services (M = 11.9), analyse government policy (M = 11.6), provide lived experience peer support or advocacy (M = 11.4) and deliver non‐health programs or services (M = 10.5).

### 3.2 Knowledge Consumption Preferences

Participants were asked to rate how they would like to access new suicide prevention knowledge, the results are shown in Table 2. Most participants preferred to access new knowledge through conferences, seminars and webinars (81%), colleagues or peers (80%), journal articles (79%), eNewsletters (73%) and websites and online forums (69%). Social media sites, particularly ‘X’ (previously known as Twitter; 7%) and Facebook (8%), were largely considered less desirable by participants, while LinkedIn (41%) was more desirable compared to other social media sites.

**TABLE 2:** Participants' preferred ways of accessing new suicide prevention knowledge.

	Very undesirable *n* (%)	Undesirable *n* (%)	Neutral *n* (%)	Desirable *n* (%)	Very desirable *n* (%)
Conferences seminars & webinars			8 (8.99)	45 (51.56)	36 (40.45)
Colleagues or peers		1 (1.12)	8 (8.99)	43 (48.31)	37 (41.57)
Journal articles	1 (1.14)	2 (2.27)	6 (6.82)	45 (51.14)	34 (38.64)
eNewsletters		4 (4.49)	12 (13.48)	52 (28.43)	21 (23.60)
Websites and online forums	1 (1.09).	8 (8.70)	14 (15.22)	53 (57.61)	16 (17.39)
Tertiary education (university courses)	2 (2.35)	7 (8.24)	24 (28.24)	36 (42.35)	16 (18.82)
LinkedIn	14 (15.56)	12 (13.33)	23 (25.56)	29 (32.22)	12 (13.33)
Research Gate	7 (8.14)	18 (20.93)	32 (37.21)	18 (20.93)	11 (12.79)
Blogs	10 (12.05)	17 (20.48)	33 (39.76)	22 (26.51)	1 (1.20)
Facebook	30 (34.88)	22 (25.58)	26 (30.23)	6 (6.98)	2 (2.33)
Twitter	39 (45.88)	22 (25.88)	17 (20.00)	7 (8.24)	

Additional open text responses indicated preferences for accessing new suicide prevention knowledge from personal experiences or direct engagement with people with a lived experience of suicide (such as via co‐design sessions); and by engaging with priority population groups (including Aboriginal and Torres Strait Islander communities and LGBTIQ+ people). Fifteen per cent of participants preferred accessing new knowledge through professional and informal networks (such as communities of practice, social media) or via academic publications.

### 3.3 Enablers and Barriers to Integrate Research Evidence Into Work

Participants were asked about enablers that supported them to use research evidence in their work. More than two thirds of participants indicated that research findings supported by lived experience (71%) and professional development (68%) were enablers. In addition, over half of the participants identified having a workplace that is open to change (54%) and processes for updating practices (51%) as an enabler. Additional enablers included having adequate resources such as time and funding, appropriate expertise, and knowledge translation resources such as plain language summaries and practical implications of research findings.

Perceived barriers to integrating research evidence in their work included limited funding and resources (56%), time constraints (52%) and evidence not being supported by people with lived experiences (37%) or not being culturally appropriate (25%), as well as workplace governance (24%). Additionally, 15% of participants identified research jargon or terminology as a barrier to applying evidence in their work. Other barriers included limited knowledge and a lack of awareness of evidence by leadership, research evidence that does not consider the local setting and its sociodemographic context, and governance limitations including risk aversion and other managerial priorities.

### 3.4 Format and Content Preferences

Most participants indicated that plain language summaries (84%) and fact sheets (78%) would be very useful translational tool formats, followed by guidelines (70%), infographics (68%), systematic reviews (68%), and frequently asked questions (64%). In contrast, videos (54%), posters or printed resources (52%), decision trees (48%) and podcasts (42%) were perceived to be less useful. Open text responses for valuable tools also included a ‘search engine’, ‘website’ or ‘central site’ that is a ‘one‐stop for information’ or that includes case studies.

Preferences for translational content were analysed based on respondents' role in suicide prevention. Two key content themes emerged as prominent preferences across a range of stakeholder groups. Information about best practices in suicide prevention was valued by roles related to lived experience and peer support advocacy, suicide prevention programs or services, funding or commissioning services, mental health programs or services, and developing or analysing government policy. Content related to lived experience perspectives was preferred by roles in lived experience and peer support or advocacy, developing or analysing government policy, and research. Other types of content highlighted unique content preferences among specific stakeholders. For example, those working in mental health programs or services frequently selected clinical or practice guidelines, while researchers frequently selected information about priority populations.

Twelve participants provided additional comments on the survey. Three of the 12 participants emphasised the importance of lived and living experiences of suicide in suicide prevention. For example, one participant described those with lived experience as ‘the experts on the subject of suicide prevention’, with another emphasising the trainable competencies that can be ‘included in various levels of structure for different purposes’. The remaining participants provided a diverse range of views on what would be useful to them including: timely and accurate data; information on the economic costs of suicide; evidence‐based programs; gatekeeper training; evidence‐based findings around patients who are in contact with the justice system or who engage in dual‐harm; and examples of how research is developed into real‐world impacts.

## 4 Discussion

The current study assessed the translational needs of stakeholders involved in Australian suicide prevention. Results of the survey offer a range of insights about the preferred ways stakeholders in Australian suicide prevention would like to access data and evidence‐based research findings. The study suggests that participants: (1) felt confident in their ability to access resources to improve their knowledge, (2) recognised that existing research evidence influenced their work, (3) preferred traditional and professional platforms for knowledge consumption such as conferences or journal articles, as opposed to social media sites and (4) identified research that is informed and supported by lived experience as a key enabler to integrate research evidence into their work.

Social media sites, particularly X (formerly Twitter) and Facebook were perceived to be less desirable platforms for accessing suicide prevention knowledge. In contrast, LinkedIn was rated as more desirable, possibly owing to its professional focus. Twitter rebranded as X in July 2023 and data collection for this study occurred between August and September 2023. There was significant public discourse in the media at that time about the future of the platform and its credibility, given that people previously barred from the site were being allowed to reactivate their accounts and a decision was made to deregulate content moderation. These findings are offset by other recent studies that examine the use of Twitter as a knowledge dissemination tool for health research [17, 18]. As such, Twitter and Facebook could still potentially be useful for disseminating information and research findings for some cohorts.

The survey results shed light on the barriers and enablers in applying research evidence to suicide prevention work. Most participants emphasised the importance of research being supported by people with lived experience as an enabler to applying research findings. This resonates with a growing recognition of the expertise of individuals with lived experiences of suicide and suicidal distress and how tools focussing on incorporating lived experience perspectives into research could be explored [19, 20]. Conversely, over one third of participants identified evidence not being supported by those with lived experience as a barrier to applying evidence.

There has also been an increased interest in embedding or including lived experience perspectives in the research process. While the benefits of this approach are largely acknowledged in the mental health sector, there are various views and often contrasting perspectives on how this should be put into practice [21, 22, 23]. VocLE and Orygen have both recently published guidelines for involving lived experience perspectives in research; however, a gap still exists in terms of realising their useful recommendations in research practice [20, 24]. The VocLE Delphi study provides valuable recommendations for the active involvement of people with lived experience of suicide in suicide research, while Orygen provides resources for involving young people with lived experience in suicide prevention research. Evidence supported by lived experience perspectives may offer suicide prevention stakeholders more confidence in applying those findings to suicide prevention activities and interventions.

In terms of the preferred content formats, most participants considered plain language summaries to be useful. Eighty‐two per cent rated plain language summaries either very useful or extremely useful, only one participant rated them ‘not at all useful’. This high rating may stem from summaries being more accessible and concise compared to journal articles and given limited resources such as time were also noted as a barrier, plain language summaries require less time investment than traditional academic journal articles. Fact sheets, which serve a similar purpose of synthesising and simplifying complex information, were considered very or extremely useful by over three‐quarters of participants. Guidelines and infographics were also considered to be useful tool formats, presenting information in a structured and visually engaging way. As 15% of respondents identified research jargon or terminology as a barrier to applying evidence to their work, plain language summaries or fact sheets could help overcome the challenge of difficult language. Dorsey, Johnson and Mbwayo [25] argue that the development and use of plain language, particularly in the area of implementation science, is an essential step to facilitate collaboration and engagement.

Translational tools should prioritise these formats, with plain language summaries of research findings playing a crucial role in supporting a broad audience of suicide prevention stakeholders to understand research implications. The open‐text responses provided by some participants offered additional insights. The mention of a ‘search engine’, ‘website’ or ‘central site’ that acts as a repository for information supports the concept of an ‘implementation’ or ‘knowledge hub’.

Preferences for translational tool content varied by participants' role in suicide prevention, reflecting the diverse knowledge needs of the suicide prevention sector. Best practices in suicide prevention were consistently selected by respondents among the top three types of content for all roles except those conducting research. Lived experience perspectives were also perceived to be widely useful, being selected by all participants developing or analysing government policy, conducting research, and most of those providing lived experience advocacy or support.

One limitation of this study is related to the generalisability of the results. Participants were recruited through Australian organisations that emphasise the translation of research findings into practice, potentially resulting in respondents with common views or practices on suicide prevention and knowledge translation. Further, the survey was promoted largely through organisations that are involved in mental health promotion and suicide prevention; contacts of these organisations may already be familiar with a range of research evidence and terminology related to suicide prevention. A broader sample or larger sample size may have offered insights into how to best communicate research findings for those who may work adjacently to mental health and suicide prevention in roles which could have a positive upstream effect. Further research may aim to include participants from different sectors to offer a broader range of insights that are applicable to a wider population. Furthermore, including more specific demographic questions may help to assess whether different ages, educational backgrounds or locational context may influence preferences. Further research that may prove beneficial could include a closer focus on how implementation of research findings could be supported across various sectors.

## 5 Conclusion

This study set out to assess the translational needs of stakeholders involved in Australian suicide prevention. Participants preferred more traditional ways of accessing new knowledge, for example, seminars, professional publications and professional networks over social media platforms. The diverse range of evidence and content preferences expressed by different groups of participants underscores the wide‐ranging needs of the suicide prevention sector. These findings suggest that translational tools need to be developed and disseminated in line with the preferences of each key stakeholder group. Key barriers to applying evidence to practice included limited funding, time and resources, and evidence that is not supported by people with lived experience.

Based on these findings, key recommendations for the development of translational tools in the field of suicide prevention include: leaning on traditional, desirable approaches to knowledge consumption, such as educational sessions or webinars, eNewsletters and website content and exploring how lived experience knowledge and expertise can be incorporated into translational tools. Translational tools must address barriers and leverage enablers to improve the application of suicide prevention evidence into practice, prioritise plain language summaries, fact sheets, guidelines and infographics, and be developed and disseminated in line with the content preferences of stakeholder groups.

## Funding

This study was conducted as part of the LIFEWAYS project with funding support from the Australian Government Department of Health and Aged Care under the National Suicide Prevention Leadership and Support Program.

## Ethics Statement

All procedures performed in studies involving human participants were in accordance with the ethical standards of the institutional and/or national research committee and with the 1964 Helsinki declaration and its later amendments or comparable ethical standards. The project was exempted from ethical review by the Hunter and New England Local Health District (authorisation number AU202308‐12).

## Consent

Informed consent was obtained from all individual participants included in the study.

## Conflicts of Interest

The authors declare no conflicts of interest.

## Data Availability

The data that support the findings of this study are available on request from the corresponding author. The data are not publicly available due to privacy or ethical restrictions.
